# How to eat an idea—A roadmap for translation and impact in plant biology

**DOI:** 10.1093/plphys/kiaf178

**Published:** 2025-05-03

**Authors:** Johnathan A Napier

**Affiliations:** Plant Sciences, Rothamsted Research, Harpenden, Herts Al5 2JQ, UK

## Introduction

As we approach the end of the first quarter of the 21st century, it seems as if there has never been a better or more exciting time to work in the field of biological research. New tools such as CRISPR-Cas genome editing combined with cheap and accurate genome sequencing are helping us to unravel genetic complexities at previously only dreamed of rates. Advances in mass spectrometry and metabolomics allow for accurate identification of chemical compounds previously undetected. In addition, the power of first machine learning and now artificial intelligence (ubiquitously referred to as AI) enables previously incomprehensible volumes of data to be sorted and mined—the most visible example being the AlphaFold programs, which can predict (with high accuracy) the structure of any protein sequence deposited in the databases. Compared with the technologies that were in play only 30 years ago, science seems to have undergone a quantum advance in terms of the tools that are widely available to the global cohort of researchers. Equally, that community is now better connected, socially and professionally, with instant access to data and publications in a fashion that bears no resemblance to the analogue and hard-copy world that those of us who started our careers in the 20th century experienced.

But simultaneously, there has probably never been a more challenging time for humankind and all the species that inhabit the planet Earth. This is predominantly driven by the currently inexorable changes in climate as a consequence of human activities such as fossil-fuel combustion and parallel actions such as massive deforestation, exploitation of nonrenewable natural resources, and pollution of the environment. Changes in the environment also drive alterations in our ability to grow and protect the crops that we rely on to feed 8 billion people, as well as expose us to new diseases as vectors adapt and exploit altered climates (Pesaresi et al. 2025).

The recent COVID-19 pandemic demonstrated that science and innovation can, when both the need and focus are intense, provide technological fixes to resolve a major crisis; in that case with the very rapid generation of vaccines and the perhaps greater logistical challenge of scale-up to meet global demands. Although COVID-19 was a very specific crisis, many different strands of research (academic, industry) from across the globe combined in an unprecedented fashion to generate the tools (diagnostic kits, vaccines, therapies) to allow societies to return, in the main, to normality. Such concerted and collaborative efforts should represent a paradigm for addressing the even greater challenges (climate change, food security, etc.) we now collectively face.

With that in mind, this article will consider how the field of plant biotechnology can contribute to responding to these challenges, specifically in terms of delivering real-world solutions—by that I mean an actual, tangible product or practice that adds (hopefully improves) to our existence and that of the planet.

Partly as a reflection of the above-mentioned golden period in the advancement of technologies, academic research in life sciences has boomed in the last few decades, including in research on plants and crops. Botany, a subject that was once considered something of an academic backwater, is now rightly recognized as fundamental to life on Earth. Without photosynthetic plants and algae, our planetary atmosphere would be radically different and unlikely to support life as we know it. Moreover, the same process of fixing carbon generates the staple foodstuffs on which the global population depends for nutrition. Given that the plant kingdom plays such a pivotal role in the continued existence of life on Earth, it is also important to recognize that (like many natural resources) it is not a something we should look to dominate—rather, we should aim to coexist with in a harmonious fashion. The challenge is how to achieve this without compromising food security for a growing global population, with this requirement and the associated paradigm defined as “sustainable intensification” (Baulcombe et al. 2009). In a more detailed extension of considering how to ensure not just sufficient calories but optimal nutrition for all 8+ billion mouths, Willett et al. (2019) proposed the Planetary Health Diet, integrating food production within the constraints of so-called Planetary Boundaries (PBs). These PBs represent both natural capital and input resources, and while the scenarios modeled by the authors continuously evolve, this study (“Food in the Anthropocene: the EAT–Lancet Commission on healthy diets from sustainable food systems”, Willett et al. 2019) should be required reading for all scientists active in the life sciences, especially those of us who are focused on combining discovery research with translational, applied outcomes.

So given that there is an urgent need to provide practical, real-world solutions to some of the many challenges facing the planet, it seems appropriate to provide a simple guide as to how to move a project from a research phase into what can be considered a “development” phase. Most likely, in previous times, the latter would have been defined as applied research and, as such, considered less important than fundamental research (which of course was the literal precursor). Fortunately, we live in slightly more enlightened times, and the need for translational activities is well-recognized and appreciated. Moreover, a burgeoning entrepreneurial subculture is now equally well established as part of the research ecosystem and a key component of the knowledge-based bioeconomy. Thus, start-up and spinout ventures are now common occurrences and, in the area of plant sciences considered here, already starting to have tangible impact (which is here defined as economic and/or societal benefits). This is a significant positive diversification in how outcomes are delivered since previously the primary (if not sole) pathway was via large multinational companies, although they should be better appreciated for their key role in developing the traits that are currently deployed at scale (Napier et al. 2019a). Equally, efforts from the public sector have been successful at delivering useful and impactful innovations in the form of GM papaya, which was resistant to papaya ringspot virus (Tripathi et al. 2007).

## Every end has a beginning

So, how do you start? First, one obviously needs a discovery that warrants further evaluation and development toward a prototype (or minimum viable product in business-speak), and, as an example, I will use the experience gained and observed from converging efforts by multiple research teams to engineer transgenic plants with the non-native capacity to synthesis omega-3 long-chain polyunsaturated fatty acids (colloquially known as omega-3 fish oils) (Napier et al. 2020; MacIntosh et al. 2021). These omega-3 oils have proven human health–beneficial properties and are key ingredients in many animal feeds (including aquafeed) but represent a diminishing natural resource (Tocher et al. 2019). For this reason, quite early in the development of GM oilseed crops, this trait became an obvious (economic, societal, environmental) target. However, unlike the GM input traits (herbicide tolerance, insect resistant) that were rapidly developed and commercialized in 1990s, so-called output traits have been much slower to advance to the same point, predominantly because they are significantly more complex in nature (Napier et al. 2019a). For example, herbicide tolerance can be conferred by a single gene, whereas traits such as omega-3 LC-PUFAs require at least 5 genes. Initial attempts to assemble the biosynthetic pathway in model systems (yeast, Arabidopsis) confirmed the functionality of the heterologous genes encoding the enzymatic activities (desaturases, elongases) and allowed for a more targeted phase of activities focused on demonstrating that these transgenes could be coordinately expressed in a tissue-specific (seed) manner, altering the seed fatty acid composition and importantly observing that these non-native fatty acids were accumulated in the storage lipid (triacylglycerols) of the seed (Venegas-Calerón and Napier 2023). From a metabolic engineering perspective, optimizing this process is fraught with jeopardy, since although the heterologous omega-3 pathway is being generated by the action of at least 5 transgenes, for the pathway to be active requires the simultaneous contribution of multiple endogenous components too (such as electron transport chain partners, acyltransferases, reductant generation, etc.). It is for this reason that this particular engineering has been likened to the trans-dominant metabolic reprogramming observed in some marine viruses (Michaelson et al. 2010). Equally, although contemporary thinking often portrays transgenesis and dependent disciplines (including engineering biology) as highly predictive and precise, this is more an aspiration than fact. In reality, plant genetic engineering still continues to teach us how little we understand the systems we are trying to manipulate (Dong and Ronald 2021).

**Figure 1.**
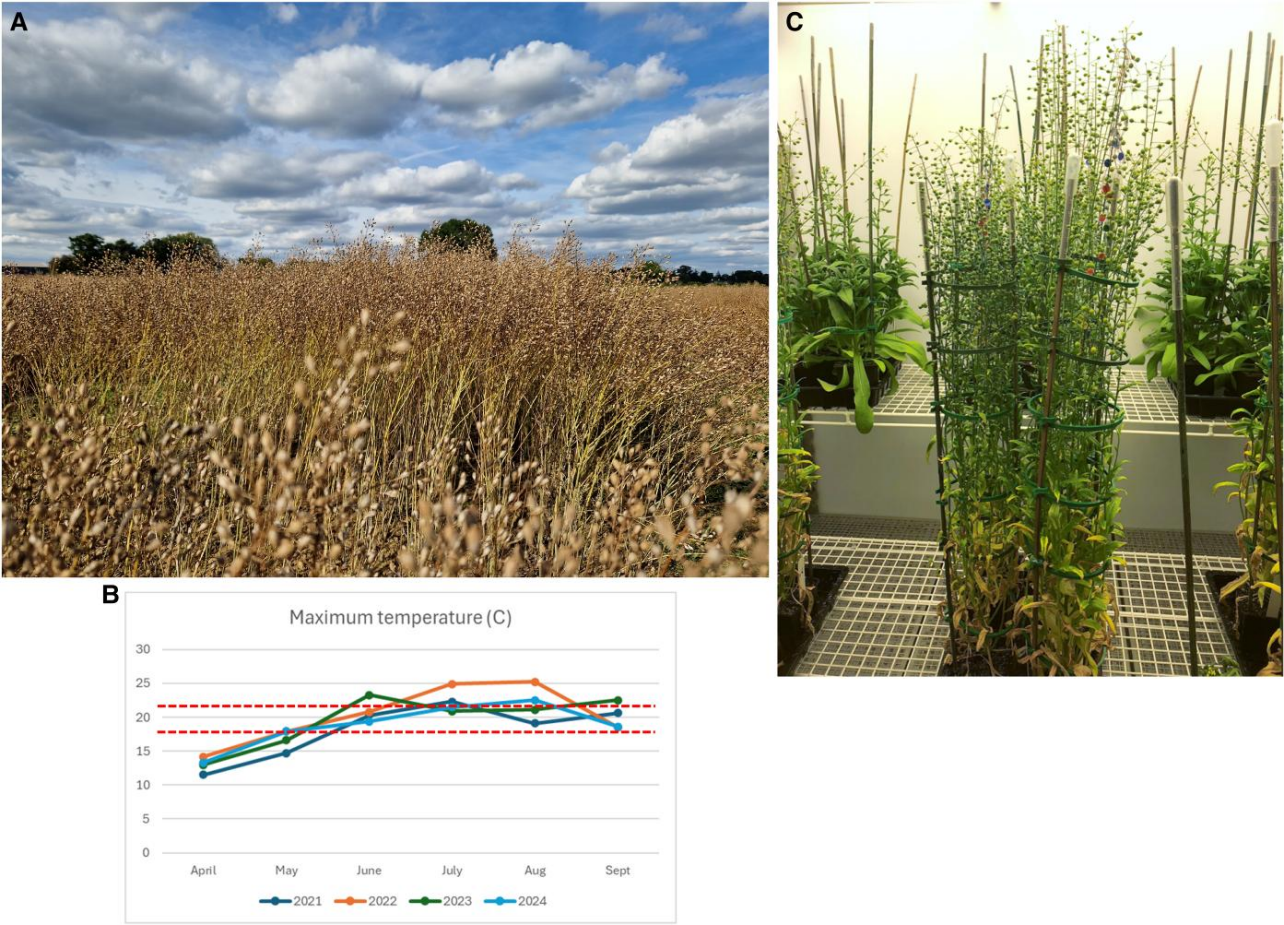
Visual comparison of Camelina growing in the field and in a controlled environment. **A)** Transgenic Camelina plants grown (under Consent 23/R8/01) on the Rothamsted Research experimental farm in Summer 2024. **B)** Maximum monthly average temperatures recorded at Rothamsted during the growing season (2021–2024)—dotted lines at temperatures referred to in (C). **C)** Similar lines grown in CE cabinets at Rothamsted Research; the set temperature is 21 ^°^/18 °C on a 16/8 day/night cycle. Note that this bears little similarity to what the crops experience in the field.

Having achieved what can be considered a proof-of-principal (PoP) (in this case, that transgenic plants can synthesize and accumulate the omega-3 fish oils EPA and DHA in their seed oils), efforts were then focused on demonstrating proof-of-concept (PoC), that is, that this innovation could stably work in the real world and at scale (Khaipho-Burch et al. 2023). In research using plants as the host, it is often assumed within the academic community (but much less frequently demonstrated) that any new discovery will be compatible with the preexisting agricultural systems and can be simply adopted in a plug-and-play fashion, irrespective of the background germplasm. Sadly, this is wishful thinking and only emphasizes the gap between fundamental plant sciences research and translational efforts using agriculture—collectively, we must strive to close this gap. Perhaps the first (and critical) step on the PoC journey is to carry out field trials to confirm the stability of the novel (GM) trait and also the ability of the modified plant to withstand the gauntlet of the variability and stresses of the natural environment. It is a seductive fallacy (enabled by experimental designs with a dependency on highly controlled environments) that results obtained in CE cabinets or glasshouses will be directly replicated in field conditions (Nelissen et al. 2020). Unfortunately, this is rarely the case (if at all), although logically it is equally unsurprising—no field has a stable temperature, month after month, with sunset and sunrise at exactly the same time each day, yet this is the paradigm that underpins much of fundamental plant sciences today (Fig. 1). So, it should not be a shock that many discoveries that showed promise in contained environments fail when they come face-to-face with cold (or hot) hard reality (Nelissen et al. 2020; Inzé and Nelissen 2022). As a first step, we as a community need to very quickly incorporate field evaluation into the DBTL (design-build-test-learn) rationales for plant engineering biology, at least for any target traits that is envisaged to be deployed at scale—this also should be costed into funding bids to enable such work and also as a clear sign of intent to funders. Given that one of the strongest arguments for using a plant chassis in engineering biology is that the preexisting know-how and infrastructure of agriculture can be harnessed to deliver massive volumes of product that would be prohibitive for any fermentation-centric chassis, it is genuinely surprising that field evaluation has not yet become a central component of plant engineering biology DBTL, unless counterintuitively, all the traits under development are not required at scale.

Although the field is the obvious destination for a plant-based innovation with potential, there are a number of real or perceived barriers to achieving this stage. First, it might be that the innovation/PoP has been constructed in a plant species that it not suitable for field evaluation. This is less common now that much research is carried out directly in crops as opposed to model systems, but a significant volume of discovery research is carried out in *Arabidopsis thaliana*. Other established or emerging engineering biology model systems such as *Physcomitrella patens*, *Marchantia polymorpha*, and *Brachypodium distachyon* have undergone limited to negligible evaluation in native conditions. While some commendable efforts were made in the past to establish protocols for transgenic Arabidopsis field trials (Frenkel et al. 2008) and also highlight the importance of field studies for understanding gene-environment interaction, such approaches gained little traction at that time with the very large Arabidopsis research community. In the past decade, in a move stimulated by funders and the desire to more quickly realize impact from fundamental activities, much more research is now carried out directly in crop species, including commodity crops such as wheat, rice, canola, and soybean, as well as more niche “boutique” crops such as camelina, pennycress, and many others. In theory, once an innovation has reached the PoP threshold in a crop, then it should be straightforward to evaluate performance in the field, for either general fitness or the specific trait of interest. Disappointingly, the number of field trials of transgenic (GM) plants remains remarkably low when set against the many hundreds if not thousands of labs generating transgenic plants. To be fair, it is often argued that most research projects are of a relatively modest duration (3–4 years), which might restrict the likelihood of being ready to move to the field. But equally, it is to be hoped that some of such projects are successful in securing a second tranche of funding to advance their research, and since this is likely because of initial success, would it not then be logical to carry out field trials? That this absence of field evaluation serves as a bottleneck for translation and technology transfer is undeniable and also represents an impediment to economic return on the initial investment that funded the research. Irrespective of whether this funding comes from private or public sectors, PoC validation by field testing is currently a missing link for many national programs, compounded by variations in the ease with which approval to carry out GM field trials might be obtained. For example, approval for environmental release in North America is straightforward and therefore more commonplace than in the UK, where the process also involves a 48-day open consultation in which members of the public (actually usually anti-technology NGOs) can make representations to the ministry responsible for approving such releases. While it is likely that this additional scrutiny can serve as an ideological impediment for some researchers and/or institutions, it can also serve a useful purpose to enhance the clarity of thinking (such as the perceived benefits) associated with a project.

However, in some parts of the world, most notably within parts of the European Union, there is strong resistance to even small-scale experimental field trials, and researchers in Italy have recently had GM trials vandalized and destroyed (Meldolesi 2024). Having myself been witness to the vandalization of GM field trials at Rothamsted in the late 1990s and also seeing first-hand how disturbing the threat of destruction can be (when our GM wheat trial in 2012 was the subject of a campaign to be “decontaminated”) (Nature editorial 2012 ), such behaviors are not conducive to a productive research culture nor a respectful debate about the pros and cons of technology. Irrespective of these impediments, it is vital for meaningful translation of any biotech innovation to undergo field trials, so mechanisms and processes need to be sought to allow scientific methods to be applied without the risk of sabotage or destruction. In the case of our GM wheat trial, the plants were engineered to constitutively synthesize the sesquiterpene (*E*)-β-farnesene, a volatile compound that is also an alarm pheromone for cereal aphids, which in turn damage plants and serve as vectors for a number of viruses. Lab-based studies identified transgenic wheat lines that emitted (*E*)-β-farnesene capable of repelling colonizing aphids as well as recruiting predatory parasitic wasps that use the alarm pheromone as a location cue (Bruce et al. 2015). Equally, using experiments carried out in growth chambers to mimic predicted field conditions confirmed these multitrophic interactions mediated by the transgene-encoded aphid alarm pheromone. However, GM field trials over 2 years revealed no statistical difference between the transgenic wheat and the controls in terms aphid repellence or parasitoid recruitment (Bruce et al. 2015). Although an associated commentary described the experiment as a “failure” (Cressey 2015), this was not a true representation of the outcome—rather (and as noted in the same piece) it was a hypothesis there to be tested. Better to know the shortcomings of the current iteration quickly so that a new one can be developed (described in Cressey [2015] as “try, try again,” but equally it could now be described as DBTL). John Pickett, the PI of the Rothamsted project, was quoted at the time as saying, “the field is the ultimate arbiter” (Cressey 2015), although it is slightly chastening that this rather obvious truism has, ten years later, still failed to gain much traction within the plant engineering biology community (Khaipho-Burch et al. 2023). However, it is equally clear that the combination of concerns over vandalism combined with ingrained aversion to risk (fear of failing) has likely impeded the translation of many discoveries. Perhaps we need to remember that nullifying the hypothesis is not “failure” but simply the scientific method in action. The reality of this is nicely documented by Simmons et al. (2021), who report a 1% success rate for the field evaluation of candidate genes in maize.

So, if we are willing to accept the proposition that the “the field is the ultimate arbiter” of the utility of a particular trait or enhancement, how can we fast-track the necessary field evaluation, especially in regions where such trials are contentious? One creative scenario has been developed in Switzerland, in the canton of Zurich, with the establishment of the so-called “Protected Site,” a dedicated field-testing capability that is available for all researchers to test their GM and GE crops in an area that is secure from intrusion or sabotage. This has allowed Swiss researchers to evaluate their technologies faster than their neighbors in France or Germany, who lack such a capability but also have strong restrictions on GM field trials. In a time when science is considered to operate without borders, it is perhaps pragmatic to consider the utility of field trials in any suitable location if it allows for initial validation of a PoP. Ultimately, creative solutions need to be found to advance exciting basic discoveries, and these must include the expansion of the DBTL process to encompass real-world performance. As a simple demonstration of the feasibility of carrying out cross-border field trials, we at Rothamsted have hosted GE camelina field trials for colleagues in France (Faure and Napier 2018) and equally carried out GM camelina trials in both Canada and the US (Han et al. 2020). So even if you are based in a location or territory that is not enabling for the field release of GM plants, this need not represent an impenetrable obstacle but rather just an opportunity to look for solutions on a wider horizon. Equally, any hesitancy that is based on a “fear of failure” should be assuaged by the realization that iteration and the DBTL cycle are dependent on identifying bugs and glitches (or even system failures); otherwise, there is no need for a recursive process. In general, one should always be scanning the horizon for collaborators who have key skills or capabilities to help advance a project. And an informal network of challenge-led, application-driven plant biotechnologists could be an additional mechanism to inject resilience into your innovation journey.

## The road less travelled—the path to a product

Although making bold claims about the utility of discovery research is now an engrained and almost mandatory component of securing funding, in reality this is poorly matched by delivery—perhaps fortuitously, there is no official mechanism by which over-claiming is called to account. And although some of this can be excused as over-enthusiasm or perhaps stretching objectives, equally some of it represents either unfamiliarity with downstream requirements or just plain hubris. Perhaps a useful default position to take is don’t believe the hype and, more importantly, don’t indulge in it either. Although not restricted to plant sciences, there is a simplistic and seductive narrative that we are “feeding the world,” which can then play well with funders and also dissemination into the wider media. I would argue that we (as a community) should be much more active in terms of self-policing ourselves in using such rhetoric, as repetition (in the absence of real step-change differences) just results in narrative fatigue and a generalized jaundiced view of research. In addition, there is a lack of familiarity within the academic research community as to the multiple steps that are required to convert a validated PoC into something that people might ultimately eat and growers would embrace (Simmons et al. 2021). Some of that might be due to the massive expansion of lab-based molecular studies with a concomitant shrinkage in field-based studies—here in the UK, many universities no longer have agricultural sciences departments or similar, so there is both a lack of experience and an absence of training opportunities. In general, there is a worrying void in knowledge and competencies in how a PoC discovery would be bulked up, deregulated, approved, and commercialized. And even if the expectation is that academic discoveries are ultimately brought to market via a public-private partnership (i.e. in collaboration with industry), how will that be achieved? Private industry is driven by the understandable need to turn a profit and answer to their shareholders and investors, and such cold logic is often a rude awakening to academics who are not normally exposed to the brutality of the market. Ultimately, if a potential product is not economically viable and stable as an inherited trait, then it does not matter how impressive the underlying science is. This is a reality that many in academia find hard to accept, and partly it then drives the “grade inflation” of the overclaiming mentioned above. But we as academics need to better understand the needs and drivers of private industry—it can be highly instructive to spend even a short period of time in that environment to better appreciate the commonalities and differences between the 2 sectors. In addition, there are other ways in which useful innovations can reach their targets (Ronald 2014).

When we at Rothamsted initially realized that we had successfully developed a prototype camelina plant that was accumulating commercially relevant levels of omega-3 long-chain polyunsaturated fatty acids, the project was greatly enabled by 2 fortuitous factors. First, the senior leadership at the time had significant experience in developing biotech traits for translation beyond academia and were familiar with the regulatory approval processes in different regions and countries (Nelissen et al. 2014). There was also a strong realization that to maximize the credibility of our prototype, not only did it have to be based on solid biological data (e.g. multiple independent transgenic events, validated by field trials), but the basis of the economic opportunity also needed to be quantified in detail and sense-checked at every step (Clarke and Zhang 2013). The importance and power of this approach has recently been highlighted by Oliveira-Filho et al. (2024), who very nicely articulate the value of these Fermi calculations in substantiating (or not) the plausibility of a particular approach. One key step forward in progressing our technology was to appoint a business development expert, with the appropriate skills in financial analysis and commercial planning. Such skills are obviously different from those of a plant biotechnologist (such as this author), but they represent key competencies that will be required in the transition from academic research through to development and ultimate commercialization (Barnes 2025). Equally important, it is critical to have a clear understanding of the regulatory requirements for bringing a GM crop to market—this is a complex process and varies from country to country, and without such approvals it will not be possible to commercialize the technology. The approval process is unfamiliar to many but usually is required for cultivation (i.e. growing the novel crop) and (separately) for use as a feed or food. It can be daunting to navigate the requirements for these approval processes, and perhaps more challenging are the costs associated with generating the data packages that are required by the national agencies that provide regulatory approval (the mechanism by which the safety of a new innovation is determined). For example, approval in the US for the commercial cultivation (referred to as deregulation, as a successful approval removes the GM event from regulated oversight) will require several years' worth of multilocation field trials, in addition to genomic and proteomic confirmation of the trait stability (Napier et al. 2019b; MacIntosh et al. 2021). Although the former is now significantly more straightforward, accurate quantitation of transgene-encoded proteins remains technically challenging (Bushey et al. 2014). Collectively, obtaining the information required for regulatory approval will likely cost several million dollars, as well as take significant time (including that required for additional field trials), and is usually restricted to an actively selected lead event. Unfortunately, this process is very often not fundable via the predominant public sector mechanisms that support the (precursor) discovery research—in other words, there is an apparent disconnect where research is advanced to a particular technology readiness level but then ineligible for further to support which might facilitate the final phase of translation and commercialization. One likely reason for this is the (logical) expectation that innovations developed in the public sector (academia) will likely need to be brought to market as a public-private partnership, that is, with private industry providing know-how and expertise on the commercial side of things (Ronald 2014; Simmons et al. 2021). However, it could be argued that this model is slightly old-fashioned and does not fully reflect the more entrepreneurial ecosystems that now exist on many university campuses and research parks. Instead, there is a need for access to experts who can provide a pragmatic analysis of what is needed to ensure regulatory approval, without this being a component of a wider business relationship—in other words, bespoke consultancy-based regulatory analysis which in turn then allows the creators to obtain additional funding (from venture capitalists, investors, etc.) because they can correctly define the costs that need to be expended to obtain deregulation. And assuming that these costs can ultimately be covered by revenue and profit from the sale of the final product—information that will have been generated by the business development expert as part of their market opportunity and business case—then it will be significantly easier to convince investors and raise the necessary funding for regulatory approval.

In my experience, we tend to consider many of the post-PoC activities we have undertaken as “derisking”—not for our technology per se but rather to reduce the apparent risk to investors and stakeholders. For example, our technology (GM camelina plants engineered to make omega-3 fish oils) has a primary market opportunity in servicing the needs of the aquaculture industry (Napier et al. 2020), which currently uses unsustainable marine extraction as a source of fish oils that are essential for marine fish farming (Tocher et al. 2019). To demonstrate that the EPA + DHA–rich camelina oils derived from our transgenic plants were suitable for use in aquaculture as a drop-in replacement for oceanic-derived fish oils, we carried out multiple fish feeding studies and confirmed the utility of our novel oil. Importantly, the camelina oil was shown to be safe and efficient as a feed ingredient for multiple different commercial fish species (Tocher et al. 2024). Although such studies are unlikely to be published in the more prestigious journals usually associated with academic success, they demonstrated successful translation of our technology and the associated derisking. Quite rightly, investors need to be reassured that any money they invest in a technology is not going to fail at an early stage of the development cycle, since they expect a return on their investment. In the case of our aquafeed trials, until we tested the suitability of our oil as a component of the diets, we were dealing with an unknown. And while one would logically predict that our GM-derived oil would be functionally identical to an oil derived from marine extraction, until this is experimentally proven it represents a risk and potential barrier to investment. It is for this reason that we have subsequently demonstrated that utility of our oil as a component of diets for salmon, trout, sea bass, and tuna, since each represents another “unknown.” But another benefit of these studies is that they represented an opportunity to bid for research funding for support that was distinct from the preceding grants. One significant challenge facing public sector–funded research can be characterized as the “cult of novelty,” where funding agencies and journals are enthralled by the concept that novelty is a justification in itself. But just because something is novel does not mean it has an intrinsic worth (academic or otherwise). Ultimately, there is a balance to be struck between advancing the project while satisfying the needs of the funding agency, but sometimes it can be useful to restate that translation can be novel too.

## Intellectual property—the patented elephant in the room

One additional consideration in any plans for the translation and commercialization must include intellectual property (IP) and patents. Although perhaps underappreciated, the patent mechanism provides a means by which an invention is protected and rewarded. In the case of publicly funded research, patents provide a key route for recovering the investments made by the taxpayer. Among academics, there is also a suspicion that patents prevent innovation, but that is likely based on a lack of familiarity with IP rights and the fact that, in general, patents actually foster innovation. One key action in planning the commercialization of a validated prototype should be to carry out detailed analysis of the patent landscape around this technology—this should also include so-called freedom-to-operate analysis, which determines if your invention is encumbered by any other IP, along with the more straightforward determination of whether your discovery is novel and patentable. Although such analysis is often undertaken by specialists (patent attorneys), it is possible for the academic researcher to carry out much of this analyses themselves, using excellent search tools such as The Lens (www.lens.org) developed by Cambia (established by Richard Jefferson, a plant biotechnologist best known for popularizing the GUS reporter system in plants). Tools such as The Lens were specifically developed to empower and enable researchers to better understand IP networks and to have greater control over their inventions; in that respect, I would strongly encourage all researchers to use them to investigate the IP surrounding their gene of interest. Often, academic researchers are surprised (and slightly horrified) to discover that “their” gene has already been the subject of a patent, but this knowledge can be empowering since it helps to crystalize an understanding of wider interest in any given technology and also provides an opportunity for transactions and business development. Perhaps surprisingly, the total patent literature is enormous, a dominant “dark matter” that the vast majority of academic researchers ignore or are oblivious of. But I would again encourage everyone to investigate the patent literature, because you will discover that it contains many advances and innovations not reported in the conventional scientific literature. Routinely searching the patent databases is obviously an absolute requirement for successful commercialization of technology, but in general, it should be an activity that is included in general good practice for conducting research; otherwise, any familiarity with the state-of-the-art will be partial and incomplete.

It is also worth emphasizing that just because something is already patented, this does not preclude others working on it or aiming to improve the invention. In fact, it is often possible to obtain new IP based on making an improvement to a system. Equally, the presence of a patent should not be seen as a block or impediment to others using the patented technology. Probably the best current example of this is CRISPR-Cas9—this technology is subject to complex IP claims and grants but is also now a universally used research tool. This also serves to emphasize the possibility of using patented inventions for research purposes (so-called research use exemptions), although these vary from region to region and are usually restricted to specific academic endeavors. In the case of the CRISPR IP, the patent owners have granted research exemptions, allowing this powerful technology to be used freely in academic research (e.g. Li et al. 2022). Obviously if technology is used to develop something useful (i.e. a new invention/prototype), then this would be dependent (encumbered) on the original foundation IP, and any commercialization would likely require a license from the owners of that IP. Some very useful lessons from the Golden Rice story are recounted by Dubock (2014) including the importance of understanding IP, even in the context of an innovation with a noncommercial focus.

Box 1: Some key considerations When considering how to develop a discovery (or even just an idea) into a product, all of the following should be given significant attention and thought.

- Have a very clear and precise vision of success—what it is that you are trying to create, and how.
- Understand the realistic timescales to achieve this success, including the constraints of seasonality—map these out.
- Analyze and investigate the IP landscapes that will almost certainly preexist and impact on your idea. Do not be put off by prior art but seek professional advice from qualified patent attorneys once you have done your own analysis.
- Understand the market opportunity that you believe underpins the economic case for the ultimate commercial success for your invention.
- With the help of a business development professional, develop a business development plan which realistically captures costs and potential returns.
- Understand the regulatory landscape that covers your technology, covering both initial translation (e.g. research GM field trials) and full commercial regulatory approval. This will likely also require specialist input from experts. Appreciate the data packages that are required for such approvals and the associated requirements in terms of time and money.
- Build a network of colleagues that share your vision and ambition. This should extend beyond the research environment and be more than an echo-chamber. This can help sense-check the direction of travel or trouble-shot a roadblock.
- Adopt an entrepreneurial mindset and relish the challenges this journey will certainly bring. Do not see knockbacks as failures but opportunities to learn.
- Keep the faith in your idea—if you do not believe in it, why should anyone else?

## Conclusions

Bringing an innovation to market is very far from the normal academic experience, but it represents the opportunity to deliver real-world impact (and all the benefit that might bring) as well as deliver personal growth (Box 1). It is for good reason that I have often described this journey as “The Road Less Travelled” (borrowing from the same title of a book by M. Scott Peck) since it is not an opportunity that is presented to all and, equally, not a road that everyone is interested in choosing to travel. But equally, and here reflecting the focus of Peck's book, I believe that actively engaging with the challenges associated with converting an idea into a tangible product is both positive and enhancing (professionally and more widely). What I have tried to outline in this short article, with the help of some examples and my own experience, are the steps that need to be taken to advance from the initial excitement of discovering something new and useful, all the way through to bringing a product to market. I am providing this with the benefit of hindsight, and if I had to rerun my omega-3 project again, I would certainly do things differently. But ultimately, we all can only do the best we can with the cards that we hold at the time. One very positive development in recent years has been to greatly encourage entrepreneurial approaches within academia, and I believe that this has also created a more robust and vibrant culture in terms of innovation, translation, and impact. It is also very encouraging to see others recently documenting their similar journeys on bringing innovations to market (Martin and Butelli 2024) Given the many challenges that we all face, this can only be a good thing, and hopefully many more people will get to literally eat our ideas.

## Dive Curated Terms

The following phenotypic, genotypic, and functional terms are of significance to the work described in this paper:

initial AmiGo: PO:0004011

## References

[kiaf178-B1] Barnes S . Building value: the business of venture capital. Wiley; 2025.

[kiaf178-B2] Baulcombe D, Crute I, Davies B, Dunwell J, Gale M, Jones J, Pretty J, Sutherland W, Toulmin C. Reaping the benefits: science and the sustainable intensification of global agriculture. The Royal Society; 2009.

[kiaf178-B3] Bruce TJA, Aradottir GI, Smart LE, Martin JL, Caulfield JC, Doherty A, Sparks CA, Woodcock CM, Birkett MA, Napier JA, et al The first crop plant genetically engineered to release an insect pheromone for defence. Sci Rep. 2015:5(1):11183. 10.1038/srep11183 26108150 PMC4480004

[kiaf178-B4] Bushey DF, Bannon GA, Delaney BF, Graser G, Hefford M, Jiang X, Lee TC, Madduri KM, Pariza M, Privalle LS, et al Characteristics and safety assessment of intractable proteins in genetically modified crops. Regul Toxicol Pharmacol. 2014:69(2):154–170. 10.1016/j.yrtph.2014.03.003 24662477

[kiaf178-B5] Clarke JL, Zhang P. Plant biotechnology for food security and bioeconomy. Plant Mol Biol. 2013:83(1–2):1–3. 10.1007/s11103-013-0097-1 23860797

[kiaf178-B6] Cressey D . GM wheat that emits pest alarm signals fails in field trials. Nature 2015. 10.1038/nature.2015.17854

[kiaf178-B7] Dong OX, Ronald PC. Targeted DNA insertion in plants. Proc Natl Acad Sci U S A 2021:118(22):e2004834117. 10.1073/pnas.2004834117 34050013 PMC8179203

[kiaf178-B8] Dubock A . The present status of golden rice. J Huazhong Agric Univ. 2014:33:69–84. https://www.goldenrice.org/PDFs/Dubock-The_present_status_of_Golden_Rice-2014.pdf

[kiaf178-B9] Editorial . Misplaced protest. Nature 2012:485(7397):147–148. 10.1038/485147b 22575919

[kiaf178-B10] Faure JD, Napier JA. Europe's first and last field trial of gene-edited plants? eLife 2018:7:e42379. 10.7554/eLife.42379 30558714 PMC6298765

[kiaf178-B11] Frenkel M, Johansson Jänkänpää H, Moen J, Jansson S. An illustrated gardener's guide to transgenic Arabidopsis field experiments. New Phytol. 2008:180(2):545–555. 10.1111/j.1469-8137.2008.02591.x 18721164

[kiaf178-B12] Han L, Usher S, Sandgrind S, Hassall K, Sayanova O, Michaelson LV, Haslam RP, Napier JA. High level accumulation of EPA and DHA in field-grown transgenic Camelina—a multi-territory evaluation of TAG accumulation and heterogeneity. Plant Biotechnol J. 2020:18(11):2280–2291. 10.1111/pbi.13385 32304615 PMC7589388

[kiaf178-B13] Inzé D, Nelissen H. The translatability of genetic networks from model to crop species: lessons from the past and perspectives for the future. New Phytol. 2022:236(1):43–48. 10.1111/nph.18364 35801919

[kiaf178-B14] Khaipho-Burch M, Cooper M, Crossa J, de Leon N, Holland J, Lewis R, McCouch S, Murray SC, Rabbi I, Ronald P, et al Genetic modification can improve crop yields—but stop overselling it. Nature 2023:621(7979):470–473. 10.1038/d41586-023-02895-w 37773222 PMC11550184

[kiaf178-B15] Li J, Scarano A, Gonzalez NM, D'Orso F, Yue Y, Nemeth K, Saalbach G, Hill L, de Oliveira Martins C, Moran R, et al Biofortified tomatoes provide a new route to vitamin D sufficiency. Nat Plants 2022:8(6):611–616. 10.1038/s41477-022-01154-6 35606499 PMC9213236

[kiaf178-B16] MacIntosh SC, Shaw M, Connelly M, Yao ZJ. Food and feed safety of NS-B5ØØ27-4 Omega-3 Canola (*Brassica napus*): a new source of long-chain Omega-3 fatty acids. Front Nutr. 2021:8:716659. 10.3389/fnut.2021.716659 34660659 PMC8514783

[kiaf178-B17] Martin C, Butelli E. The purple tomato story; from laboratory bench to the consumer. ACS Food Sci Technol. 2024:5(1):19–28. 10.1021/acsfoodscitech.4c00692 39840404 PMC11744742

[kiaf178-B18] Meldolesi A . Italy tests first gene-edited vines for winemaking. Nat Biotechnol. 2024:42(11):1630. 10.1038/s41587-024-02478-8 39506141

[kiaf178-B19] Michaelson LV, Dunn TM, Napier JA. Viral trans-dominant manipulation of algal sphingolipids. Trends Plant Sci. 2010:15(12):651–655. 10.1016/j.tplants.2010.09.004 20934366

[kiaf178-B20] Napier JA, Haslam RP, Olsen RE, Tocher DR, Betancor MB. Agriculture can help aquaculture become greener. Nat Food. 2020:1(11):680–683. 10.1038/s43016-020-00182-9 37128041

[kiaf178-B21] Napier JA, Haslam RP, Tsalavouta M, Sayanova O. The challenges of delivering genetically modified crops with nutritional enhancement traits. Nat Plants 2019a:5(6):563–567. 10.1038/s41477-019-0430-z 31160704

[kiaf178-B22] Napier JA, Olsen RE, Tocher DR. Update on GM canola crops as novel sources of omega-3 fish oils. Plant Biotechnol J. 2019b:17(4):703–705. 10.1111/pbi.13045 30485634 PMC6419714

[kiaf178-B23] Nelissen H, Moloney M, Inzé D. Translational research: from pot to plot. Plant Biotechnol J. 2014:12(3):277–285. 10.1111/pbi.12176 24646295

[kiaf178-B24] Nelissen H, Sprenger H, Demuynck K, De Block J, Van Hautegem T, De Vliegher A, Inzé D. From laboratory to field: yield stability and shade avoidance genes are massively differentially expressed in the field. Plant Biotechnol J. 2020:18(5):1112–1114. 10.1111/pbi.13269 31587443 PMC7152599

[kiaf178-B25] Oliveira-Filho ER, Campos-Silva R, Hanson AD. Running Fermi calculations as a superpower to gauge reality. Plant Physiol. 2024:kiae347. 10.1093/plphys/kiae347 Online ahead of print. 38876095

[kiaf178-B26] Pesaresi P, Bono P, Corn S, Crosatti C, Daniotti S, Jensen JD, Frébort I, Groli E, Halpin C, Hansson M, et al Boosting photosynthesis opens new opportunities for agriculture sustainability and circular economy: the BEST-CROP research and innovation action. Plant J. 2025:121(3):e17264. 10.1111/tpj.17264 39910851 PMC11799749

[kiaf178-B27] Ronald PC . Lab to farm: applying research on plant genetics and genomics to crop improvement. PLoS Biol. 2014:12(6):e1001878. 10.1371/journal.pbio.1001878 24915201 PMC4051633

[kiaf178-B28] Simmons CR, Lafitte HR, Reimann KS, Brugière N, Roesler K, Albertsen MC, Greene TW, Habben JE. Successes and insights of an industry biotech program to enhance maize agronomic traits. Plant Sci. 2021:307:110899. 10.1016/j.plantsci.2021.110899 33902858

[kiaf178-B29] Tocher DR, Betancor MB, Sprague M, Olsen RE, Napier JA. Omega-3 long-chain polyunsaturated fatty acids, EPA and DHA: bridging the gap between supply and demand. Nutrients 2019:11(1):89. 10.3390/nu11010089 30621155 PMC6356973

[kiaf178-B30] Tocher DR, Sprague M, Han L, Sayanova O, Norambuena F, Napier JA, Betancor MB. Inclusion of oil from transgenic Camelina sativa in feed effectively supplies EPA and DHA to Atlantic salmon (Salmo salar) grown to market size in seawater pens. Food Chem. 2024:456:139414. 10.1016/j.foodchem.2024.139414 38901077

[kiaf178-B31] Tripathi S, Suzuki J, Gonsalves D. Development of genetically engineered resistant papaya for papaya ringspot virus in a timely manner: a comprehensive and successful approach. Methods Mol Biol. 2007:354:197–240. 10.1385/1-59259-966-4:197 17172756

[kiaf178-B32] Venegas-Calerón M, Napier JA. New alternative sources of omega-3 fish oil. Adv Food Nutr Res. 2023:105:343–398. 10.1016/bs.afnr.2023.01.001 37516467

[kiaf178-B33] Willett W, Rockström J, Loken B, Springmann M, Lang T, Vermeulen S, Garnett T, Tilman D, DeClerck F, Wood A, et al Food in the Anthropocene: the EAT-Lancet commission on healthy diets from sustainable food systems. Lancet 2019:393(10170):447–492. 10.1016/S0140-6736(18)31788-4 30660336

